# Analysis of region**-**specific changes in gene expression upon treatment with citalopram and desipramine reveals temporal dynamics in response to antidepressant drugs at the transcriptome level

**DOI:** 10.1007/s00213-012-2714-0

**Published:** 2012-05-01

**Authors:** Magdalena Gąska, Maciej Kuśmider, Joanna Solich, Agata Faron-Górecka, Małgorzata J. Krawczyk, Krzysztof Kułakowski, Marta Dziedzicka-Wasylewska

**Affiliations:** 1 Department of Pharmacology, Institute of Pharmacology Polish Academy of Sciences, Smętna 12 Street, 31-343 Krakow, Poland; 2 Faculty of Physics and Applied Computer Science, AGH University of Science and Technology, al. Mickiewicza 30, 30-059 Kraków, Poland

**Keywords:** Antidepressant, Desipramine, Citalopram, Gene expression, RT-PCR, In situ hybridization, Time-course, Delayed onset of action, Rats

## Abstract

**Rationale:**

The notion that the onset of action of antidepressant drugs (ADs) takes weeks is widely accepted; however, the sequence of events necessary for therapeutic effects still remains obscure.

**Objective:**

We aimed to evaluate a time-course of ADs-induced alterations in the expression of 95 selected genes in 4 regions of the rat brain: the prefrontal and cingulate cortices, the dentate gyrus of the hippocampus, and the amygdala.

**Methods:**

We employed RT-PCR array to evaluate changes during a time-course (1, 3, 7, 14, and 21 days) of treatments with desipramine (DMI) and citalopram (CIT). In addition to repeated treatment, we also conducted acute treatment (a single dose of drug followed by the same time intervals as the repeated doses).

**Results:**

Time-dependent and structure-specific changes in gene expression patterns allowed us to identify spatiotemporal differences in the molecular action of two ADs. Singular value decomposition analysis revealed differences in the global gene expression profiles between treatment types. The numbers of characteristic modes were generally smaller after CIT treatment than after DMI treatment. Analysis of the dynamics of gene expression revealed that the most significant changes concerned immediate early genes, whose expression was also visualized by in situ hybridization. Transcription factor binding site analysis revealed an over-representation of serum response factor binding sites in the promoters of genes that changed upon treatment with both ADs.

**Conclusions:**

The observed gene expression patterns were highly dynamic, with oscillations and peaks at various time points of treatment. Our study also revealed novel potential targets of antidepressant action, i.e., *Dbp* and *Id1* genes.

**Electronic supplementary material:**

The online version of this article (doi:10.1007/s00213-012-2714-0) contains supplementary material, which is available to authorized users.

## Introduction

Despite the increasing prescription of antidepressant drugs (ADs) and their intensive investigation for almost five decades, the mechanisms of their antidepressant action remain unresolved. Some major hypotheses related to depression include monoamine deficiency (Elhwuegi 2004), abnormalities in the hypothalamus–pituitary–adrenal (HPA) axis, and the cytokine hypothesis (Leonard and Myint 2009), but the nature and involvement of these components in the etiology of depression remains uncertain. In addition to the monoamine deficiency hypothesis, depression is often associated with the sustained hyperactivity of the stress axis caused by the stress neurotransmitters, corticotrophin-releasing hormone (CRH) and urocortin, the effects of which are mediated through the CRHR1 and CRHR2 receptors (Leonard and Myint 2009). There is also evidence that inflammatory processes play an important role in depression, and most antidepressants have anti-inflammatory effects (Leonard and Myint 2009; Maes et al. 2009). Preclinical and clinical evidence suggest that neuropeptides (such as galanin, neuropeptide Y, and proenkephalin) and their receptors may be directly involved in depression and related illnesses (Dziedzicka-Wasylewska and Papp 1996; Dziedzicka-Wasylewska et al. 2002; Kuteeva et al. 2008; Redrobe et al. 2002).

Based on the above-mentioned data and literature survey (Boehm et al. 2006; Carr et al. 2010; Drigues et al. 2003; Frechilla et al. 1998; Orsetti et al. 2008; Urani et al. 2005), genes of interest that are believed to be crucial in ADs action were selected for our study. These genes encode receptors (e.g., *Adra*, *Adrb*, *Cnr*, *Drd*, *GalR*, *Gria*, and *Opr*), transcription factors (e.g., *Creb*, *Crem*, *Egr*, *Fos*, *Jun*, *Jund*, and *Sp1*), genes involved in inflammatory responses (e.g., *Il10r*, *Il1r*, *Il6r*, *Stat*, and *Tnf*), HPA activity (e.g., *Crhbp*, *Crhr*, and *Ucn*), neuronal apoptosis and synaptic plasticity (e.g., *Bcl2*, *Bdnf*, *Cacna*, *Cdk*, *Ntf*, and *Vgf*), and a number of other genes previously unlinked directly to ADs action but important because of their involvement in the transcriptional machinery of the cell (e.g., *Aes*, *Arnt*, *Cebpb*, *Dbp*, *Esr2*, *Ets*, *Gata2*, *Hdac*, *Id1*, *Nr*, *Pax6*, and *Yy1*). In contrast to the vast majority of preclinical studies, which have been conducted using animals treated with ADs repeatedly (typically 7–14 days), we decided to conduct a temporal analysis of the expression of genes regulated by ADs in specific brain regions.

The notion that the onset of ADs action generally takes weeks is widely accepted. However, questions concerning the sequence of molecular events that are necessary for the therapeutic effects to occur during the time following drug administration remain unanswered. This delay in response suggests that antidepressant action is a complex multicellular process that requires the orchestration of many biochemical events, including changes in the transcription of genes that may not be directly involved in the neurotransmitter signaling cascade. Previous studies have shown that delayed onset of ADs action (e.g., changes in receptor density) is induced not only by different types of chronic treatments but also by single-dose antidepressant treatments followed by a certain period of drug-free time (Dziedzicka-Wasylewska and Rogoz 1997; Dziedzicka-Wasylewska et al. 2001; Kuśmider et al. 2006, 2007). Therefore, we additionally checked whether a single dose of a drug followed by a drug-free period induced changes similar to those observed after repeated AD treatments.

Because the activation of the limbic system is a relatively consistent biological finding in patients with major depression (aan het Rot et al. 2009; Hercher et al. 2009; Ressler and Mayberg 2007; Tanti and Belzung 2010), we focused our study on the time-dependent effects of ADs on the expression of selected genes in the limbic regions of the brain, including the prefrontal cortex (PCX), the cingulate cortex (CCX), the dentate gyrus of the hippocampus (HIP), and the basolateral nucleus of the amygdala (AMY).

Therefore, we applied automated laser microdissection, RNA isolation, and amplification (custom RT-PCR arrays) to determine the expression level of selected genes and to investigate the mode of action of the tricyclic antidepressant desipramine (DMI) and selective serotonin reuptake inhibitor citalopram (CIT) in the above-mentioned four regions of the rat brain. The laser microdissection technique allowed the precise excision of a defined population of cells, and the RT-PCR arrays allowed the quantification of minute amounts of transcripts. Additionally, we used an advanced bioinformatics approach, i.e., singular value decomposition (SVD) representation of time-dependent multiple gene expression data, which allowed the comparison of global gene expression patterns across different brain regions and used ADs. The SVD is an analytic procedure which has been successfully adopted to analyze the time dynamics of multiple gene expression datasets (Holter et al. 2000; 2001; Simek et al. 2004).

Through the experimental design of our study, we expected to highlight some unresolved questions regarding the molecular mechanisms of antidepressant action. First, what is the time-course of antidepressant action, i.e., which molecular targets are affected by a specific AD and at what time during treatment does it occur? What are the structure-specific changes in gene expression that occur during ADs treatment? Are there common end points of antidepressant action, i.e., do ADs belonging to different pharmacological classes target a final common molecular pathway? Finally, does acute treatment followed by a drug-free period of time induce similar molecular changes to those caused by repeated treatment?

## Methods

### Animals

Male Wistar rats weighting 250–310 g at the beginning of the experiments were used. Animals were housed six to eight per cage under a 12-h dark/light cycle, with food and water supplied ad libitum. All animal procedures used in this study were approved by the local Bioethics Commission at the Institute of Pharmacology, Polish Academy of Sciences (Krakow, Poland).

### Drug treatment

**Fig. 1:**
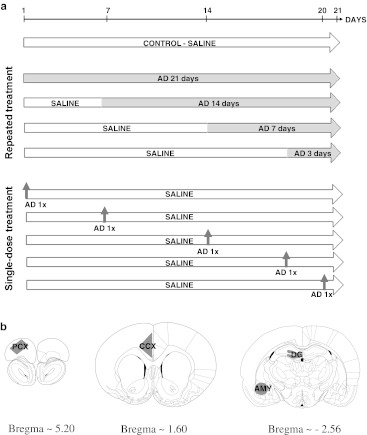
(**a**) A schema of the experimental procedures used. The experiments lasted for a total of 21 days. The control groups received saline for 21 days. The repeatedly treated groups were treated either with DMI or CIT for 21, 14, 7, or 3 days. The single-dose treatment groups received drugs only once during the experiments at the same time points (21, 14, 7, and 3 days and one additional day before the end of the experiment), followed by a drug-free period of time and saline injections. (**b**) Drawings of the coronal diagrams adapted from the rat brain atlas (Paxinos and Watson 1998) and the microdissected brain regions in which gene expression was studied, i.e., the prefrontal cortex (*PCX*), the cingulate cortex (*CCX*), the dentate gyrus of the hippocampus (*HIP*), the basolateral nuclei of the amygdala (*AMY*)

DMI and CIT were administered in doses of 15 mg/kg in a volume of 2 ml i.p. daily, and all animals were injected once a day during the 21 consecutive days of the experiment, either with saline or antidepressant. The doses of drugs were chosen based on our previous experience and literature data (Kuśmider et al. 2006, 2007; Finnegan et al. 1987; Nibuya et al. 1995, 1996; Zhao et al. 2008; Fujimaki et al. 2000; Thome et al. 2000; Castagné et al. 2009). The control groups received vehicle (saline) for 21 days, and four groups were treated repeatedly with the appropriate drug for 21, 14, 7, and 3 days prior to the end of the experiments. There were additional groups treated with a single dose of AD and sacrificed at the same time points (i.e., 21, 14, 7, and 3 days) and 1 day afterwards. The experimental paradigm is presented in Fig. 1a. Brains were removed 24 h after the last injection, rapidly frozen in a heptane/dry ice mixture, and kept at −80°C until preparation.

### Tissue collection and RNA isolation

Brains were always sectioned by the same investigator into 50-μm slices using a cryostat (Leica Microsystems GmbH). Neuroanatomical descriptions are based on the nomenclature provided by the rat brain atlas (Paxinos and Watson 1998). Four brain regions were excised with a laser microdissector (Leica Microsystems GmbH): the PCX, the CCX, the HIP, and the AMY (for the coronal diagrams and microdissected areas, see Fig. 1b). A MicroRnase Kit (Qiagen Inc.) was used to extract total RNA from the microdissected samples. The total RNA concentration was measured using a NanoDrop ND-1000 Spectrometer (ThermoScientific Inc.). The quality of the isolated RNA was checked using a microcapillary electrophoresis system (BioRad) according to the manufacturer’s instructions, and samples that passed the quality threshold (RIN > 8.0) were used for further experiments.

### Quantitative RT**-**PCR

mRNA was reverse transcribed with random hexamers to generate single-stranded cDNA transcripts according to the manufacturer’s protocol of the High Capacity cDNA Archive Kit (Applied Biosystems). For qPCR reactions, cDNA from three individuals was taken for each group. A predesigned TaqMan Low Density Array (TLDA) in a 96-well format (Applied Biosystems) was used, and duplicates were performed for the expression of each gene in each sample (for the accession numbers of the probes used, see Supplementary file 1). A total amount of 100 ng of cDNA for each sample, RNase-free water, and 50 μl of TaqMan Universal PCR Master Mix were loaded into each reservoir to a total volume of 100 μl/reservoir. Real-time PCR amplifications were performed using a 7900 HT Real-Time PCR System (Applied Biosystems). The thermocycling conditions for the reactions were a 10-min initial setup at 95°C, followed by 40 cycles of 30 s denaturation at 97°C and 1 min annealing/extension at 60°C. The results for each plate were analyzed using ABI PRISM SDS2.3 (Applied Biosystems) software to calculate the raw value of Cq (quantification cycle, also known as the threshold cycle, crossing point, or take-off point) in each well. 18 S rRNA was used as an endogenous control, and Rpl32 and Ppia were used as reference genes. The levels of expressed genes were measured using a relative quantitative method (Pfaffl 2001), with the Ppia and Rpl32 genes as reference genes and a control group (saline treated) as a calibrator (Applied Biosystems, UserBulletin#2).

### Data analysis

Genes whose Cq values were higher than 38 or undetermined in more than a half of the samples per analyzed brain region were excluded from analysis (for each structure and each drug, there were 10 time groups, *n* = 6 per group). Statistical analysis was performed on the list of probes detected in each structure after each specific drug treatment. All statistical analyses of the qPCR data used ∆Cq values (Cq normalized to endogenous controls), whereas graphs represented the fold change (2−∆∆Cq) ± SEM. Data were analyzed by two-way analysis of variance (ANOVA) with the treatment type-specific factor (single dose or repeated) and time factor. Correction for multiple testing was applied separately for each of the main factors from the ANOVA by controlling for the percentage FDR (Benjamini and Hochberg 1995). ANOVA was followed by a Bonferroni test as a post hoc analysis to determine where significant differences occurred. The threshold level for statistical significance was *p* < 0.05.

### Gene ontology analysis

The functional annotation analysis tool DAVID 2008 was used to identify over-represented gene ontology (GO) groups among the gene expression profiles and to group the genes into functional classifications (Huang et al. 2009). The lists of gene symbols (Entrez IDs), together with a particular background annotation distribution (sets of genes for which mRNA levels were detected in each brain structure), were used as the input files. Over-represented GO terms (GOTERM_CC_ALL, GOTERM_BP_ALL, GOTERM_MF_ALL) were defined as having at least three transcripts and a maximum EASE score of 0.1.

### Identification of transcription factor binding sites enriched in coregulated transcripts

Identification of over-represented transcription factor binding sites (TFBSs) was performed using the cREMaG database (Piechota et al. 2010) by submitting Entrez IDs lists of genes that reached statistical significance for each ANOVA factor. Functional promoter sequences were identified by carrying out alignments between the 5′ upstream sequences of orthologous rat and human genes. The analysis was performed with the default parameters of a 70 % conservation threshold and a maximum number of 10 most conserved TFBSs in noncoding regions between 5,000 bp upstream and 1,000 bp downstream of the transcriptional start site.

### SVD analysis

Data on multiple gene expression at sequential time points were analyzed using the SVD as a means to capture dominant trends, called characteristic modes. SVD was performed for the data, which were prepared as follows: first, the whole dataset was subjected to ANOVA (Brandt 1997). The aim of this procedure was to select a set of genes for which the changes during our experiments were statistically significant. For each time series, the value *F*_c_ was calculated:

$$ {{F_{\text{c}}} = \frac{{s_{\text{A}}^2}}{{s_{\text{W}}^2}}} $$

where:

$$ {s_{\text{A}}^2 = \frac{1}{{t - 1}}\sum\limits_{{i = 1}}^t {{n_i}} {{\left( {{{\bar{x}}_i} - \bar{x}} \right)}^2}}, $$

and

$$ {s_{\text{W}}^2 = \frac{1}{{n - t}}\sum\limits_{{i = 1}}^t {\sum\limits_{{j = 1}}^{{{n_i}}} {{{\left( {{x_{{ij}}} - {{\bar{x}}_i}} \right)}^2}} } } $$

In our case, *t* is the number of time points and *n* is the total number of samples for a given time series. Here, we assumed that, if for a given time series ${{F_{\text{c}}}/{F_{\text{t}}} \geqslant 1.5}$ (where *F*_c_ is the value calculated for this time series and *F*_t_ is the theoretical value for the significance level equal to 0.9), it was considered for further analysis. Next, the filtered time series were normalized (Stahlberg et al. 2008). This procedure reduces the length of each time series by one, as the value of the expression without any treatment is used as the reference value. The value at each other time point is calculated in accordance with the following formula:

$$ {A{E_i} = \frac{{{\text{d}}{E_i} - \left\langle {{\text{d}}E} \right\rangle }}{\text{SD}}}, $$

where d*E*_*i*_ is calculated as ${{\text{d}}{E_i} = {{\log }_2}\left( {{2^{{{E_0} - {E_i}}}}} \right)}$; *E* is the expression at a given time point *i*; $\left\langle {{\text{d}}E} \right\rangle$ is the mean; and SD is the standard deviation of the expression at a given time point.

Mathematically, SVD means that the *n* × *m* matrix **A** is represented as${A = US{V^T}}$ where *U* is an *n* × *n* matrix; *V* is an *m* × *m* matrix; and *S* is an *m* × *m* diagonal matrix of eigenvalues of matrix **A**. Our interest lies in the number of positive eigenvalues, *r*, on which basis we are able determine the so-called characteristic modes of matrix **A**, such that $$ {X = \left[ {\begin{gathered} {X_1} \\ \vdots \\ {X_r} \\ \end{gathered} } \right] = \left[ {\begin{gathered} {s_1}{v_1} \\ \vdots \\ {s_r}{v_r} \\ \end{gathered} } \right]} $$.

In the case of a gene expression time series, each row of matrix **A** represents the expression profile of one gene and each column represents the level of expression of each gene at a given time point. Here, SVD was performed if the number of genes selected in the ANOVA was greater than the number of time points, i.e., 5 (1, 3, 7, 14, and 21 days). The expression profile of any given gene is a linear combination of the obtained modes with coefficients equal to the corresponding elements of the matrix **U**. The contribution of each mode to the expression profile of each gene can also be calculated (Simek et al. 2004).

### In situ hybridization studies

The brains of animals treated with CIT for 3 days repeatedly or in single dose and from the control group were stored at –80°C. The frozen brains were cut into 12-μm slices using a cryostat (Leica). The following antisense oligonucleotide probes complementary to the mRNAs encoding the selected genes were used: c-*fos*, 5′-GCA GCG GGA GGA TGA CGC CTC GTA GTC CGC GTT GAA ACC CGA GAA CAT-3′ (Curran et al. 1987); Egr1, 5′-CCG TTG CTC AGC AGC ATC ATC TCC TCC AGT TTG GGG TAG TTG TCC-3′ (Milbrandt 1987); Nr4a1, 5′-GGT GGC AGG TGC GGT GGG CGC CGT CTC GGG GCT GGC CAG GTC CAT-3′ (Hazel et al. 1988); and Id1, 5′-GCA CTG CTA CTG GCG ACC TTC ATG ATC CTG AGG ACA GGC GGA GAG G-3′ (designed and checked for specificity in BLAST, NCBI). All probes were synthesized by Genomed (Poland) and labeled with [^35^S]dATP (Hartmann Analytic) at the 3′ end using terminal transferase (New England Biolabs). Following the preincubation, the brain slices were incubated with hybridization buffer containing the labeled probes (10,000 CPM/μl) for 18 h at 37°C, then were washed in 1× SSC solution, three times for 20 min with 2× SSC containing 50 % formamide (Merck) at 42°C and after that in 1× SSC (15 min), water (5 min) and rinsed in 70 % ethanol. The images were obtained using FujiFilm BAS 5000 Phosphoimager and they were analyzed using FujiFilm software (Image Gauge, V4.0). The identification of brain structures was defined according to the rat brain atlas (Paxinos and Watson 1998). Statistical analysis was carried out by two-way ANOVA and Student’s  *t* test was used as post hoc test to compare changes in mRNA level in CCX between the 3-day-treated rats and control group. A value of *p* < 0.05 was considered to be significant.

## Results

### SVD analyses revealed differences in the global gene expression profiles between treatment types

For each drug and brain region, two time series gene expression datasets were compared (from the repeated and single-dose modes of treatment). Time series consisting of six time points (0, 1, 3, 7, 14, and 21 days of treatment) were analyzed by SVD. We show that highly complex sets of gene expression profiles can be represented by a small number of “characteristic modes” that capture most of the expression information in the temporal patterns of gene expression change. Other aspects of the decomposition indicate the extent to which a gene exhibits a pattern similar to those provided by the singular vectors. Thus, once a set of interesting patterns has been identified, genes can be ranked by their relationship with given patterns.

**Fig. 2:**
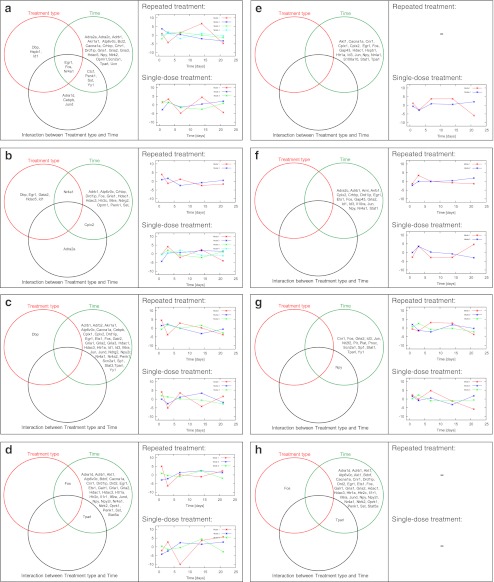
The *left* parts of the pictures include Venn diagrams comparing changes in gene expression depending on the drug and the brain region. Venn diagrams present genes for which changes in expression reached the level of statistical significance estimated by two-way ANOVA following FDR correction for multiple comparisons after DMI (**a**–**d**) or CIT (**e**–**h**) treatment in the PCX (**a**, **e**), the CCX (**b**, **f**), the HIP (**c**, **g**), and the AMY (**d**, **h**). Genes in *circles* represent significant changes in gene expression by each factor (treatment type and time), as well as their interaction. The complete results of the two-way ANOVA and *p* values are presented in Supplementary files 1 and 2. The *right* part of each picture present comparative SVD analysis of structure-dependent gene expression following repeated and single-dose DMI and CIT treatments. The datasets obtained from repeatedly and acutely treated rats were transformed separately by SVD after DMI (**a**–**d**) and CIT (**e**–**h**) treatment for each brain region: the PCX (**a**, **e**), the CCX (**b**, **f**), the HIP (**c**, **g**), and the AMY (**d**, **h**). No modes could be generated for the PCX after repeated CIT treatment and for the AMY after repeated and single-dose CIT treatment because the number of genes selected in ANOVA for a specific treatment type was smaller than the number of the time points. The genes for which the expression profiles were most similar to the global SVD mode are listed in Table 1

The overall results of the SVD analysis (SVD modes) are presented in Fig. 2. Distinct modes following repeated and single-dose treatments were detected. The contribution of characteristic mode to the expression profile of each gene was calculated, and genes with the highest contribution to each mode are listed in Table 1. The number of genes whose expression profiles were best described by a characteristic mode were larger for the single-dose treatment. The global temporal change in gene expression following repeated DMI treatment in the PCX was dominated by the top four modes, whereas that following the single-dose treatment was dominated by the top three modes (Fig. 2a, right part). The mode sets for the CCX, the HIP, and the AMY are presented in Fig. 2b–d, right part. These figures revealed a complex pattern of expression, with oscillations at different time points of treatment. The temporal patterns of gene expression after DMI treatment described by the use of modes varied among the brain regions and between treatment types (repeated and single dose). However, there were some genes that could be grouped together (described by a common mode), although their temporal expression profiles differed between treatment types (the modes that contributed the most to their expression have different time-courses). After CIT treatment, the numbers of characteristic modes were generally smaller than after DMI treatment. No modes could be generated for the PCX after repeated CIT treatment and for the AMY after repeated and single-dose CIT treatment because the number of genes selected in ANOVA for a specific treatment type was smaller than the number of the time points.

**Table 1:** Genes with expression profiles most closely approximating the SVD modes after treatment with DMI (top) and CIT (bottom) in the PCX, CCX, HIP, and AMY

Region	Treatment	Mode	Gene symbol
Desipramine			
PCX	DMI repeated	Mode 1	Akr1a1, Bcl2, Cacna1a, Crhbp, Ctcf, Egr1, Gria2, Hdac1, Id1, Jund, Ntrk2, Penk1, Ppia, Scn2a1, Sst, Stat3, Tparl,Yy1
		Mode 2	Adra2a, Cebpb, Cplx2, Dbp, Gria3, Pir
	DMI single-dose	Mode 1	Adra1d, Adra2a, Arnt, Atp6v0c, Cnr1, Creb1, Crhbp, Crhr1, Ctcf, Egr1, Gria1, Gria2, Npy, Ppia, S100a10, Sp1, Sst, Tparl, Yy1
		Mode 2	Fos
		Mode 3	Nr4a1, Penk1
CCX	DMI repeated	Mode 1	Adrb1, Arrb2, Dbp, Hdac1, Hdac3, Id1, Penk1
		Mode 2	Adra2a, Cplx2
	DMI single-dose	Mode 1	Atp6v0c, Cplx2, Crhbp, Drd1ip, Egr1, Fos, Gria1, Htr2c, Nr4a1, Sst
		Mode 2	Adrb1, Arrb2, Hdac1, Penk1
		Mode 3	Adra2a, Hdac5
HIP	DMI repeated	Mode 1	Adra1d, Adrb1, Akr1a1, Arnt, Atp6v0c, Dbp, Egr1, Ets1, Gria2, Hdac3, Npy2r, Ppia, Stat3
		Mode 2	Cacna1a, Fos, Id1, Penk1
		Mode 3	Bcl2, Nr4a1
	DMI single-dose	Mode 1	Adrb1, Atp6v0c, Cdk5, Cplx2, Crhbp, Drd1ip, Egr1, Ets1, Galr2, Gria2, Gria3, Hdac3, Htr1a, Id3, Il1r1, Jun, Jund, Ndrg2, Npy2r
		Mode 2	Akt1, Galr3, Npy
		Mode 3	Id1
AMY	DMI repeated	Mode 1	Adrb1, Aes, Akr1a1, Atp6v0c, Cdk5, Cebpb, Crhbp, Crhr1, Gria1, Gria2, Gria3, Hdac3, Hdac5, Nr2f2, Ntrk2, Ppia
		Mode 2	Akt1, Fos, Nr4a1, Nr4a2, Oprk1
	DMI single-dose	Mode 1	Adra2c, Adrb1, Aes, Akr1a1, Akt1, Arrb1, Atp6v0c, Bcl2, Bdnf, Cacna1a, Cdk5, Cnr1, Crhr1, Dbp, Drd1ip, Egr1, Ets1, Fos, Galr1, Gap43, Gria3, Hdac1, Hdac3, Htr2c, Id1, Il1r1, Il6, Jund, Nr4a1, Ntrk2, Oprk1, Pax6, Penk1, Pomc, Rxra, S100a10, Scn2a1, Sst, Tparl
		Mode 2	Hdac5, Ndrg2, Nr4a2, Ppia
		Mode 3	Gria1, Gria2, Id3, Ntf3, Stat5a
Citalopram			
PCX	CIT single-dose	Mode 1	Adrb2, Cdk5, Cnr1, Ets1, Fos, Gap43, Hdac1, Hspb1, Htr2c, Id1, Id3, Il6ra, Nr4a1, Pax6, Ppia, Rxra, S100a10, Stat1
		Mode 2	Cplx1
CCX	CIT repeated	Mode 1	Egr1, Fos, Nr4a1
		Mode 2	Ntf3, S100a10, Stat1
	CIT single-dose	Mode 1	Adra2c, Arnt, Drd2, Egr1, Ets1, Fos, Galr2, Gata2, Htr2c, Id1, Id3, Jun, Nr4a1
		Mode 2	Arrb2, Drd1ip, Esr2, Grip2
HIP	CIT repeated	Mode 1	Id3, Ndrg2, Npy, Plat, Pnoc, Ppia, Scn2a1, Stat1, Tparl, Yy1
		Mode 2	Arrb2, Galr3
		Mode 3	Ets1, Npy2r
	CIT single-dose	Mode 1	Ets1, Fos, Gria3, Htr1a, Id1, Id3, Il6ra, Jun, Nr2f2, Nr4a1, Plat, Pnoc, Rxra, Sp1, Stat1
		Mode 2	Drd2, Sst

Datasets were sorted by the administered drug and brain region. Gene expression values for each time point (0, 1, 3, 7, 14, and 21 days) were arranged into a time series specific for each treatment type (repeated and acute). Genes for which the expression profiles over time most closely approximated the global expression (SVD modes) are listed for DMI (top) and CIT (bottom) treatment

### Comparisons between drug and brain regions indicated multiple time**-**dependent changes in gene expression

The strategy of a detailed time-course study of gene expression alterations upon antidepressant treatment was applied to verify the question whether acute and repeated administration of an AD results in similar effects. To analyze the dynamics of the changes and the influence of the time and treatment type-specific factors, two-way ANOVA was performed. The genes for which expression changes reached the level of statistical significance (*p* < 0.05 for each factor after multiple comparison corrections) are presented in Venn diagrams in Fig. 2a–h, left part. The ANOVA was followed by the analyses of the biological function of the obtained gene sets by examining over-representation of GO terms and TFBSs (Table 2).

**Table 2:** Functional GO classes and TFBSs over-representation in groups of genes associated with time-dependent changes in expression after the DMI (top) and CIT (bottom) treatments

Region	GO		TFBS over-representation		
	GO term	Fold changes (*p* value)	Binding site	TFBS class	Fold change (*p* value)
Desipramine					
PCX	GO:0015075~ion transmembrane transporter activity	3.36 (0.007)	Pax4 (MA0068)	PAIRED-HOMEO	10.1 (0.0000)
	GO:0005216~ion channel activity	3.36 (0.022)	MEF2A (MA0052)	MADS	5.2 (0.0223)
	GO:0005624~membrane fraction	2.14 (0.026)	SRF (MA0083)	MADS	4.9 (0.0317)
	GO:0016021~integral to membrane	1.51 (0.039)			
	GO:0007610~behavior	1.64 (0.049)			
CCX			MEF2A (MA0052)	MADS	5.6 (0.0037)
			Pax5 (MA0014)	PAIRED	5.6 (0.004)
			SRF (MA0083)	MADS	5.4 (0.0055)
			ESR1 (MA0112)	NUCLEAR RECEPTOR	4.6 (0.0254)
			FOXF2 (MA0030)	FORKHEAD	4.3 (0.0357)
			CEBPA (MA0102)	bZIP	4.2 (0.0452)
HIP	GO:0043234~protein complex	1.77 (0.002)	MEF2A (MA0052)	MADS	4.9 (0.0053)
	GO:0022857~transmembrane transporter activity	2.71 (0.007)	CREB1 (MA0018)	bZIP	4.9 (0.0055)
	GO:0046983~protein dimerization activity	1.86 (0.015)	SRF (MA0083)	MADS	4.6 (0.0095)
	GO:0015075~ion transmembrane transporter activity	2.71 (0.020)	Pax4 (MA0068)	PAIRED-HOMEO	4.2 (0.0225)
	GO:0042803~protein homodimerization activity	2.37 (0.022)			
AMY	GO:0004888~transmembrane receptor activity	1.85 (0.001)	SRF (MA0083)	MADS	5.4 (0.0028)
	GO:0004872~receptor activity	1.68 (0.002)	Pax5 (MA0014)	PAIRED	4.8 (0.0091)
	GO:0016021~integral to membrane	1.70 (0.002)	CREB1 (MA0018)	bZIP	4.5 (0.0161)
	GO:0004871~signal transducer activity	1.52 (0.005)	MEF2A (MA0052)	MADS	3.9 (0.0484)
	GO:0051240~positive regulation of multicellular organismal process	1.83 (0.029)			
	GO:0007610~behavior	1.60 (0.043)			
	GO:0050896~response to stimulus	1.24 (0.047)			
Citalopram					
PCX			SRF (MA0083)	MADS	7.1 (0.0005)
			MEF2A (MA0052)	MADS	5.1 (0.0191)
			Pax4 (MA0068)	PAIRED-HOMEO	4.9 (0.0232)
			CREB1 (MA0018)	bZIP	4.9 (0.0234)
CCX			SRF (MA0083)	MADS	5.6 (0.0066)
			CREB1 (MA0018)	bZIP	4.9 (0.0223)
			Pax5 (MA0014)	PAIRED	4.8 (0.026)
			MEF2A (MA0052)	MADS	4.7 (0.0289)
HIP	GO:0043234~protein complex	1.52 (0.002)	Pax4 (MA0068)	PAIRED-HOMEO	4.7 (0.0038)
	GO:0043170~macromolecule metabolic process	1.27 (0.007)	SRF (MA0083)	MADS	4.1 (0.0165)
	GO:0031981~nuclear lumen	1.62 (0.013)	CREB1 (MA0018)	bZIP	3.8 (0.0317)
	GO:0044260~cellular macromolecule metabolic process	1.25 (0.021)			
	GO:0005634~nucleus	1.24 (0.022)			
	GO:0043231~intracellular membrane-bounded organelle	1.16 (0.026)			
	GO:0005654~nucleoplasm	1.60 (0.037)			
	GO:0060255~regulation of macromolecule metabolic	1.20 (0.041)			
AMY			MEF2A (MA0052)	MADS	11.7 (0.0000)
			CREB1 (MA0018)	bZIP	6.1 (0.0195)
			NFKB1 (MA0105)	REL	5.7 (0.0281)
			SRF (MA0083)	MADS	5.5 (0.0371)
			ESR1 (MA0112)	NUCLEAR RECEPTOR	5.2 (0.0474)

The statistical significance of GO enrichment for the time-dependent changes in gene expression was computed using algorithms implemented in the DAVID 2008 database. The analyses were performed on lists of genes that correspond to changes in gene expression that reached the level of statistical significance after two-way ANOVA for the time factor. An empty chart report means that there were no annotations that passed the specified threshold (maximum EASE score, 0.05). For TFBS searches, the cREMaG software was used. The fold change of the detected number of identified TFBSs compared to the number expected by chance and the statistical significance of the over-representation of the TFBS-containing genes compared to the number expected by chance were computed using a *z* score test

### Functional analyses of genes exhibiting significant changes during the treatment period and the identification of transcription factor binding sites

GO terms were identified separately for each dataset (two-way ANOVA filtered), specific brain region and drug, according to the observed temporal gene expression changes (*p* < 0.05 for the time factor after correction for multiple comparisons). The results for each dataset are presented in Table 2.

To find molecular factors involved in the transcriptional control of the observed gene expression changes over time, an in silico method of TFBS identification was used. We analyzed gene promoters on the assumption that a subset of the genes with altered expression following a specific treatment might be coregulated by common transcription factors. For this purpose, we used the cREMaG database. The results are presented in Table 2. Surprisingly, the over-representation of binding sites was similar between brain regions and between drugs. After DMI treatment, the TFBSs for myocyte enhancer factor 2 (MEF2A) and serum response factor (SRF) were over-represented. In addition to SRF and MEF2A, PAX5, ESR2, FOXF2, and CEBPA factors were also over-represented in promoters of genes with altered expression in CCX and CREB1 factor was over-represented in the HIP and AMY. Following CIT treatment, the over-represented TFBSs partly overlapped with those identified for DMI treatment in the same brain regions, with the SRF, MEF2A, and PAX families of transcription factors being affected in both cases. Interestingly, the CREB1 binding site was over-represented in all brain structures after CIT treatment.

### Dynamics in gene expression at the level of individual genes

**Fig. 3:**
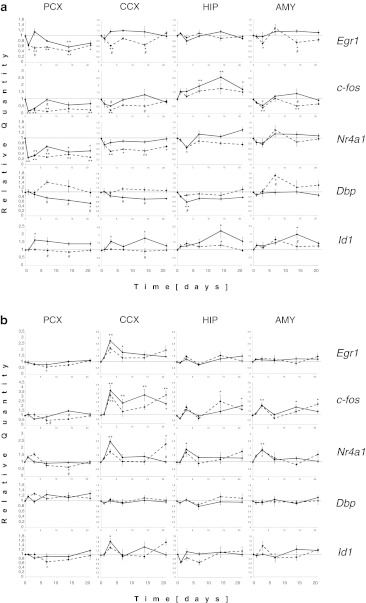
Individual gene expression profiles for some transcription factors after (**a**) DMI and (**b**) CIT treatments. Values represent the relative transcript levels (±SEM). *Solid line* repeated treatment, *dashed line* single-dose treatment. For the lists of genes with significant changes in expression estimated by ANOVA according to the time factor, the treatment type factor, or the interaction between the two factors, see the Venn diagrams (Fig. 2). *p < 0.05, **p < 0.01 vs control; #*p* < 0.05 difference between repeated and single-dose treatments

The analyses described above revealed the diversity in the global gene expression profiles depending on the brain structure and the drug used. Although they provide an overall view, they give no answer about the time-course of single, specific genes. We have chosen a subset of genes for which expression was significantly altered according to two-way ANOVA. Figure 3 presents the changes in the mRNA levels of certain transcription factor genes for which the observed changes were the greatest and were statistically significant in almost all regions. Interestingly, a high correlation was observed in the expression profiles between *Fos* and *Nr4a1* and, to a lesser extent, *Egr1*. In the PCX, CCX, and AMY, the initial decrease in *Fos* mRNA was observed, with the subsequent increase at day 7, compared to the control level. Differences between the repeated and single-dose treatments were apparent. Similar changes were observed for *Nr4a1* in the PCX and CCX. Significant differences between the repeated and single-dose DMI treatments were also observed for the *Dbp* and *Id1*. In the PCX and CCX, there was an expression peak for *Id1* on day 3 in the PCX and CCX and on day 14 of the repeated treatment in the HIP and AMY, respectively, while its expression remained constant under the single-dose treatment. Opposite changes were observed for the *Dbp* gene for which expression constantly decreased during the repeated DMI treatment, whereas the expression level was constant for the single-dose treatment. CIT induced entirely different changes in the expression of *Fos* and *Nr4a1*, e.g., the significant increases in the mRNA levels for these genes in the CCX and almost no differences between time-courses in gene expression after repeated and acute treatment were observed. In the CCX, HIP, and AMY, there was a peak in gene expression on day 3.

Not all of the changes in the expression profiles presented in Fig. 3 are described in detail, they were meant only to show that the changes in expression for a subset of individual genes (which reached the statistical threshold of significance) occurred at different time points. Some of the profiles were similar when comparing the repeated and single-dose treatments or brain regions, while others differed.

### Evaluation of *Egr1*, c-*fos*, *Nr4a1*, and *Id1* mRNA levels by an in situ hybridization

**Fig. 4:**
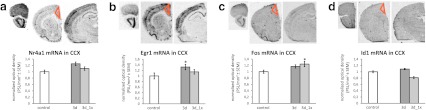
Example images of Nr4a1 (**a**), Egr1 (**b**), c-*fos* (**c**) and Id1 (**d**) mRNA expression as assessed by ISH (*up*) and quantification results for mRNA expression of these genes in the CCX in groups treated for 3 days with CIT repeatedly and in a single dose (*down*). Data represent the mean optical density±SEM (PSL/mm^2^) normalized to the control. Student’s *t* test was used as post hoc test to compare the 3-day-treated groups vs the control group; *p < 0.05

As a second, independent analysis of gene expression changes induced by AD treatment, we used in situ hybridization (ISH) to compare the regulation of genes with the most pronounced changes after CIT treatment. ISH also allowed us to evaluate potential changes in other brain areas. The mRNA encoding *Egr1*, c-*fos*, *Nr4a1*, and *Id1* was measured in the CIT-treated animals. Since the fold change of expression after CIT treatment were the most significant in CCX after 3 days, this brain region was selected for quantification at that specific time point to validate changes obtained in RT-PCR experiments. A modest but consistent increase (~20 %) in mRNA encoding *Egr1*, *Nr4a1*, and c-*fos* was observed after 3 days of repeated treatment in CCX. Overall two-way ANOVA (factors: time/treatment) revealed significant effect of time on *Egr1* (*F*_1,22_ = 5.42, *p* = 0.0295) and c-*fos* (*F*_1,14_ = 11.51, *p* = 0.004) mRNA level. In case of *Nr4a1* (*F*_1,21_ = 3.52, *p* = 0.0746) and *Id1* (*F*_1,12_ = 2.67, *p* = 0.1283), the effect of time on mRNA level did not reach the level of significance, although in both cases, the increase after 3 days of repeated treatment was observed (Fig. 4).

## Discussion

Studies based on microarray analyses of gene expression following antidepressant treatment usually compare region-specific gene expression changes either after different ADs treatment (Altar et al. 2004; Boehm et al. 2006; Conti et al. 2007; Drigues et al. 2003; Jungke et al. 2011; Knuuttila et al. 2004; Palotas et al. 2004; Ploski et al. 2006; Sillaber et al. 2008; Takahashi et al. 2006; Wong et al. 2004) or in different animal models of depression (Andrus et al. 2010; Bergstrom et al. 2007; Orsetti et al. 2008; Surget et al. 2009). In our study, we broadened the perspective by evaluating the time-course of gene expression changes during antidepressant treatment. Additionally, in our study, we applied RT-PCR arrays which have significant advantages in specificity and sensitivity compared with whole-genome cDNA microarrays whose results may often be ambiguous due to technical reasons (Ostrowski and Wyrwicz 2009).

The present study indicates that two ADs of different pharmacological profiles influenced the expression of different genes, although some common changes induced by these drugs were also observed. For the DMI treatment, there was more similarity in the inter-region comparison than in the inter-drug comparison of the same brain structures.

Recently, Lee et al*.* (2010) studied the effects of three ADs in different time points (1, 3, and 7 days) in the hippocampus. Specific gene expression patterns depending on the treatment duration have not been clearly identified, although some of the alterations reported were also observed in our study, i.e., *Arrb1*, *Cdk5*, *Sst*, and *Jund*. Changes in the expression of *Hspb1*, c-*fos*, *Crhbp*, *Egr1*, *Npy*, glutamate ionotropic receptors, *Pax6*, and members of the ATPase Ca^2+^ transporter family observed in our study were also reported by Conti et al*.* (2007) following electroconvulsive shock (ECS), sleep deprivation, and fluoxetine.

Some evidence for the importance of ion transport in the rat brain cortex have been reported in the context of antidepressant treatment (Knuuttila et al. 2004; Palotas et al. 2004). Specifically, changes in calcium channels (*Cacna* and *Cacnb*) and voltage-gated sodium channels (*Scn11a*) were observed after 28 days of treatment with amitryptiline (Boehm et al. 2006). In the present study, a change in *Gria1–3*, *Scn2a1*, and *Cacna1a* gene expression also occurred. *Gria1* and *Gria3* encode the subunits GLUR1 and GLUR3 of the glutamate ionotropic receptor (AMPA), which are implicated in the mechanism of action of ADs (Ampuero et al. 2010; Du et al. 2004; Knuuttila et al. 2004; Martinez-Turrillas et al. 2002).

The important feature of ADs treatment is the delayed onset of therapeutic effects (Machado-Vieira et al. 2010). However, some reports indicate that treatment with selective serotonin reuptake inhibitors (SSRIs) is associated with symptomatic improvement by the end of the first week or even as quickly as within the first 3 days of treatment (Taylor et al. 2006; Mitchell 2006; Katz et al. 2004). These clinical observations could reflect changes on the molecular level shortly after the initiation of therapy. The results of the present study clearly showed that expression changes of most affected genes occurred in the first 3 days of treatment after both drugs. Such immediate changes in gene expression were apparent not only when individual genes were analyzed but also in a global view of gene expression in the SVD diagrams. The observed gene expression patterns were highly dynamic, with oscillations and peaks occurring at different time points. Such temporal fluctuations during ADs treatment may be a reason for the discrepancies of results available from the literature which were often obtained at different time points of treatment. DMI, which influences noradrenaline reuptake, is known to change the expression of mRNA encoding the noradrenaline transporter in the rat locus ceruleus in a biphasic manner with a decrease after 7 days and an increase after 14 days of treatment (Zhu et al. 2002). Such biphasic changes are also seen for SSRI treatment of rats, with a temporary reduction of serotonin transporter mRNA after 7 days but not 21 days of treatment with fluoxetine (Neumaier et al. 1996). Additionally, changes in other cellular components (not directly linked to the monoamine reuptake inhibition) are also described by time-dependent fluctuations of either mRNA or protein levels. For example, the level of the glutamate ionotropic receptor GRIA2/3 reaches a peak after 7 and 21 days, but not 14 days, of treatment with DMI (Martinez-Turrillas et al. 2002), and the levels of the transcription factor AP-2 and its DNA-binding activity are decreased after 7 days, but not 21 days, of treatment with CIT (Berggard et al. 2003). There is also upregulation of glucocorticoid receptor in the hippocampus by 2 and 14 days, but not 7 days, exposure to tricyclic antidepressants (Okugawa et al. 1999). Expression of BDNF after ADs is another example: the significant decrease in expression occurs after 4 days of fluoxetine, while chronic administration of ADs induces increased BDNF expression (de Foubert et al. 2004).

From these data, it is clear that dependence of various measures on the duration of ADs treatment has already been noticed, however, not dwelled upon any further, especially in the context of any compensatory adaptations.

Analyses performed in the present work indicated an interesting pattern of expression of immediate early genes (IEGs: c-*fos*, *Egr1*, and *Nr4a1*). *Egr1* (also known as *zif268*, *Krox-24*, and *NGFI-A*) has been extensively used as a marker of neuronal responses to sensory stimuli. Despite the fact that changes in the expression of c-*fos* and *Egr1* are both considered to reflect neuronal activity, their expression patterns after acute ADs treatment differ between brain regions (Slattery et al. 2005), similar to our study. Chronic (6 or 9 days) ECS or treatment with different ADs downregulate the induction of c-*fos* mRNA in response to acute restraint stress in rat frontal cortex (Morinobu et al. 1995). A similar observation is made for *Egr1* after ECS or repeated tranylcypromine or imipramine in PCX (Park et al. 2011). In our study, we also observed downregulation of *Egr1* and c-*fos* mRNA levels in PCX at different time points during DMI treatment.

Another correlation observed in our study concerned the expression profiles of c-*fos* and *Nr4a1*. *Nr4a1* (*NGFI-B*, *Nur77*) is a member of a subgroup of nuclear steroid receptors that do not have any recognized ligands and function independently as orphan receptors with transcriptional regulatory activity. Pharmacological studies indicate that NGFI-B mRNA and protein levels are elevated via the activation of adrenergic receptors (Humphries et al. 2004).

Evaluation of mRNA level by ISH in groups of animals treated with CIT confirmed the upregulation of *Egr1*, *Nr4a1*, and c-*fos* mRNA level after 3 days of repeated treatment in the CCX. But the changes observed by ISH were not so pronounced as evaluated by an RT-PCR method. The difference in mRNA fold change may reflect the technical differences and sensitivity of these two methods used to evaluate the gene expression. Additionally, both of these methods are semiquantitative, so the most important is the direction of observed changes, not the absolute value. In gene expression studies, RT-PCR is the most sensitive method available, while ISH allows evaluating and visualizing the localization of transcripts in various other brain regions at the same time (Bartlett 2002), so they both can be considered as complementary.

As has been previously reported, the activation of IEGs, associated with synaptic activity, learning, and memory, is under the control of the SRF (Knoll and Nordheim 2009). Interestingly, our analysis of TFBSs revealed an over-representation of SRF binding sites in the promoters of genes that changed over time after both the DMI and CIT treatments in all brain regions. Also, binding sites for MEF2A, which belongs with SRF to the MADS-box transcription factors family, were over-represented. Although these transcription factors have different DNA binding properties and are required for opposing cellular processes (Schlesinger et al. 2011), an interaction between them has been recently predicted by bioinformatics approaches and confirmed experimentally (Pustylnik 2011).

One of the most interesting changes in gene expression found in this study concerned the *Dbp* gene, which showed a progressive decrease during repeated DMI treatment. The D site of the albumin promoter (albumin D-box)-binding protein, also known as DBP, belongs to the clock genes and is expressed according to circadian rhythms (LopezMolina et al. 1997). As sleep disturbances are common in major depressive disorders and sleep deprivation is one possible treatment for depression, *Dbp* may be involved in antidepressant action. *Dbp* has been already identified as a potential candidate gene in bipolar disorder and alcoholism by Niculescu and colleagues (Niculescu et al. 2000; Rodd et al. 2007; Le-Niculescu et al. 2008). According to our present study, *Dbp* may also be an interesting target for studies on the mechanisms underlying antidepressant action.

Interestingly, the repeated and single-dose treatments resulted in opposite regulation of the *Id1* gene, which was increased upon repeated DMI treatment. *Id* (inhibitor of differentiation) functions as a negative regulator of differentiation during development. ID1, together with ID2 and ID3, increases the self-renewal and proliferation potential of the cortical neural stem cells (Jung et al. 2010). In the present study, we have shown, for the first time, alterations in *Id1* expression after antidepressant treatment. Neuroplasticity is believed to be involved in ADs action, so changes in *Id1* gene expression in the brain cortex may be another potential target for further studies concerning ADs and depression.

In addition to the evaluation of temporal changes in gene expression and comparison of the effects of two ADs, the aim of our study was to verify the hypothesis that single-dose treatment induces similar molecular changes to those seen under repeated treatment. This hypothesis fits into the idea of time-dependent sensitization (TDS) (Antelman and Gershon 1998; Antelman et al. 2000) which assumes that the therapeutic effects of a drug may not be due to the drug’s action per se but they rather develop as a function of time. A drug is viewed as a foreign substance and stressor that induces a time-dependent cascade of molecular events, irrespective of whether it is given repeatedly or only once. There are many pharmacological examples of TDS (Antelman et al. 2000). In the case of ADs, the increased level of monoamines at the synapse may only be an early step in a potentially complex cascade of molecular events that results in an AD’s therapeutic effects (Pineyro and Blier 1999). The results of the present study cannot definitely confirm nor reject the hypothesis of TDS in ADs action. Interestingly, the expression of most affected genes was observed to change with time (time factor in a two-way ANOVA) but not with the treatment type, especially after CIT treatment. As described in the “Introduction” section, the genes chosen for investigation in the present study are either strongly believed, on the basis of available data, to be involved in ADs action or are novel targets for new ADs therapies. The changes in expression of many of these genes did not show any significant difference between treatment types (repeated or single-dose, followed by the same period of time). This may confirm the role of time in ADs treatment as more important than the role of the drug itself, although the two drugs used in our study activated different molecular processes and pathways, as revealed by our bioinformatics analysis. On the other hand, if we discard the TDS hypothesis, we have to question the role of many of the selected genes as true antidepressant targets since their expression was altered irrespective of treatment type.

However, one might also consider the alterations in various gene expression induced by a single dose of a given AD as compensatory mechanisms which are triggered in the brain in response to external perturbing stimuli (e.g., drug administration). One may also suppose that such mechanisms are triggered each time the drug is administered, resulting in complex, reciprocal interactions at many levels, also in gene expression—therefore, different alterations are observed at various time points.

The methodology employed here enables to detect very subtle changes in gene expression, which is especially important in neuroscience (Mirnics et al. 2001). Laser microdissection allowed the precise excision of the specified populations of cells, and the RT-PCR arrays allowed us to investigate many genes at once. Because gene expression levels were measured in four regions of the same brains, inter-regional comparisons of the expression profiles and the finding of correlations between alterations in groups of genes and interconnected brain regions were possible. Data on multiple gene expression at sequential time points (0, 1, 3, 7, 14, and 21 days) were analyzed using SVD as a means to capture dominant trends, called characteristic modes. In this way, the data were represented by a small number of dominant modes which capture many essential features of the time pattern in gene expression. SVD analysis provided a global picture of the dynamics of gene expression and enabled the comparison of the expression of different genes across various brain regions upon treatment with different ADs.

The expression patterns of the investigated genes depended on the drug (DMI or CIT), drug treatment schedule (repeated or acute), and the brain region studied, which strongly indicate the specificity of the observed molecular changes upon ADs treatment and confirm the biological significance of our results. Some gene expression changes were common to both drugs used, but finding a final common pathway influenced by these two ADs was not possible. Surprisingly, considerable periodic changes in the expression levels of many genes correlated with different time points in ADs treatment, which broadens our understanding of ADs mechanism of action.

## Conclusions

In conclusion, our objective was to study the alterations of gene expression with respect to time, antidepressant used, and brain region. Appropriate genes (95) were not chosen randomly but—as we clearly stated in the “Introduction” section—the choice was based on the data already published, indicating their either proved or alleged importance for the mechanism of action of antidepressants. We decided to measure alterations in their expression in the same experiment in order to avoid any variables. What we have found is the complex picture of changes in gene expression, the picture which may help to understand discrepancies often encountered in the literature dealing with AD action. The main finding of our work is that the gene expression pattern during ADs treatment is highly dynamic, with oscillations and peaks occurring at different time points of treatment. Additionally, our study revealed a number of novel potential targets for antidepressant therapy, such as the *Dbp* and *Id1* genes, that were not previously directly linked to antidepressant action. We are strongly convinced that the time has come to look at the mechanisms of action of ADs through a broader window instead of sticking to any particular measure, as, e.g., one gene or one protein, and building new hypothesis which will lead us to the next dead end.

A similar conclusion has been put forward recently by Kessler et al. (2011), who have analyzed the results from neuroimaging studies of depressed patients. Despite the great effort, the outcome of these studies has been “a surprising heterogeneity of results with the eventual lack of practical conclusions.” The authors suggest novel approaches using longitudinal designs of neuroimaging studies, i.e., to “take various pictures over the course of treatment.” Our studies, although preclinical, strongly support that view.

## Electronic supplementary material

A table listing TaqMan Assay IDs on TLDA array (with gene symbols and their descriptions) (Supplementary file 1) and two tables listing the results of the two-way ANOVA followed by FDR correction. The lists include the genes altered by treatment type, time, and interaction between the factors for specific drugs and structures (Supplementary file 2).

ESM 1 (XLS 32 kb)

ESM 2 (XLS 97 kb)
